# Performance of a universal PCR assay to identify different *Leishmania* species causative of Old World cutaneous leishmaniasis

**DOI:** 10.1186/s13071-020-04261-5

**Published:** 2020-08-27

**Authors:** Mahmoud Nateghi Rostami, Fatemeh Darzi, Mahin Farahmand, Mohsen Aghaei, Parviz Parvizi

**Affiliations:** 1 Laboratory of Host-Parasite Interactions, Department of Parasitology, Pasteur Institute of Iran, Tehran, Iran; 2 Qom University of Medical Sciences, Qom, Iran; 3 Molecular Systematics Laboratory, Department of Parasitology, Pasteur Institute of Iran, Tehran, Iran

**Keywords:** *Leishmania*, PCR, ITS, Diagnosis, Cutaneous leishmaniasis

## Abstract

**Background:**

The characterization of *Leishmania* species is important for clinical management of the diseases and the epidemiological control of the parasite distribution. Most of the published polymerase chain reaction (PCR) amplification methods lack the ability to identify all species complexes, have low performance and usually need post-PCR procedures. There is a need for improving the diagnosis of CL by development of an accurate affordable PCR method to identify all *Leishmania* species in clinical specimens.

**Methods:**

We designed an optimized a PCR amplifying the internal transcribed spacer 2 sequence of the ribosomal RNA gene (ITS2) aligned from different strains of CL-causing *Leishmania* species in the Old World. The performance of the method was evaluated on lesion samples from several CL suspected patients and the limit of detection (LOD) was determined on DNA of promastigotes from reference strains.

**Results:**

The universal PCR enabled simultaneous discrimination of *L. major*, *L. tropica* and *L. infantum* using one primer pair in one reaction. Stained smear microscopy and Novy-MacNeal-Nicolle (NNN) medium culture alone detected 77.27% (17/22) and 72.73% (16/22) of the positive CL samples, respectively. The PCR assay showed 100% sensitivity (22/22) (95% CI: 84.56–100%) and 100% specificity (3/3) (95% CI: 29.24–100%) for species identification on isolates from lesion scraping/exudate and 100% sensitivity (13/13) (95% CI: 75.29–100%) and 100% specificity (11/11) (95% CI: 71.51–100%) for species identification on biopsy samples of CL patients, while being capable to successfully amplify as little as 0.01–0.1 pg of *Leishmania* DNA from cultured promastigotes.

**Conclusions:**

We present a validated easy-to-use affordable universal PCR assay to identify the most common Old World *Leishmania* species causing CL. This PCR assay could be used as a sensitive/specific technique to diagnose CL-causing *Leishmania* species in clinical samples with high accuracy.

## Background

Cutaneous leishmaniasis (CL) is usually manifested as a nodule which gradually develops to a self-healing lesion leaving a scar, but a polymorphism is seen in lesion characteristics, and diverse atypical forms are reported [1]. The host’s immune response, *Leishmania* species, and inter- and intra-species genetic diversity of *Leishmania* might be involved in this clinical polymorphism [2, 3]. CL is a geographically extensive disease and in the Old World can be caused by any of the four different species: *Leishmania major* causing self-healing zoonotic CL (ZCL); *L. tropica* causing anthroponotic CL (ACL); *L. infantum* principally a VL-causing species which cause CL; and *L. aethiopica* causing CL which is limited in distribution in the African region [1, 4, 5]. CL due to *L. infantum* is a sporadic disease in Asia and the Middle East where the main causes of CL are *L. major* and *L. tropica*. However, *L. infantum* CL is more frequently present in the Mediterranean Basin area including countries of North Africa and southwestern Europe. Also, in the Americas there is increasing evidence that CL due to *L. chagasi* (identical to *L. infantum*) is present, although it is more associated with VL [6].

During the last few decades, CL has geographically been extended beyond the areas where it was previously recorded [5]. The presence of multiple *Leishmania* species with overlapping clinical features that sometimes leads to misdiagnosis in endemic areas [7], as well as the genetic heterogeneity of the parasite [8, 9], accentuate requirement for development of laboratory tests with high accuracy to be used for species identification of *Leishmania* spp. [10]. However, conventional parasitology methods have an intermediate diagnostic sensitivity on dermal aspirate samples of CL, as they are influenced by the sampling procedure, type of skin lesion, parasite load and technical personnel expertise [11]. Molecular methods based upon the polymerase chain reaction (PCR) amplification of *Leishmania* DNA have been widespread for diagnosis of the causative species of CL [12–15]. Many such PCR assays have been reported in the literature, but most of the PCR amplification protocols have low accuracy to identify all species complexes, have no validity testing on clinical samples and some of them need post-PCR procedures such as sequencing, restriction digestion or melting curve analysis, which are not readily available in remote locations [16, 17].

PCR amplifications targeting the kinetoplast and ribosomal RNA genes are amongst the most commonly used approach for the diagnosis and/or identification of *Leishmania* species [18], and the internal transcribed spacer region of the ribosomal DNA repeat unit (ITS2) has previously been exploited for Old World *Leishmania* species discrimination [19, 20]. In most eukaryotic organisms, genes encoding *18S*, *5.8S*, and *28S* rRNA are organized as tandem repeats which are transcribed together, generating a long primary transcript. 5′ and 3′ external transcribed spacers (ETSs) and internal transcribed spacers (ITSs) are removed from the primary transcript by snoRNAs and multiple enzymes [21]. The *L. major* genome contains only ~12 copies of the rRNA gene repeat per haploid genome, organized in tandem arrays on chromosome 27 [22]. Due to the lack of whole genome sequencing data, the copy number of rRNA gene clusters is not clearly available for *L. tropica* and *L. aethiopica* species. Regarding *L. infantum*, analysis of available bioinformatics databases shows that different subunits of the rRNA gene are located in different chromosomes and are not arranged in a head-to-tail tandem array. The number of rRNA gene sets in another VL causing species (*L. donovani* haploid genome) is reported to be 166 [23].

The aim of the present study was to design a universal PCR method based on the ITS rDNA region to identify parasite species directly from clinical samples or *Leishmania* isolates. It is estimated that several copies of rRNA gene repeats exist in the diploid genome of different *Leishmania* species, ranging from 20 to more than 150, which makes it a good target for analyzing low parasite quantities [23, 24]. In this study, we describe a universal PCR based on ITS2 rDNA for the discrimination of *L. tropica*, *L. major* and *L. infantum* by using one pair of primers. Identification of more than one agent in one reaction is more cost-effective, more rapid, and more acceptable for the patients. We show the validation of our results on clinical samples from CL patients.

## Methods

### Study population, ethical considerations and sampling

Patients with clinical manifestations compatible with active CL who were referred to known centers for diagnosis of CL were included. The same samples which were collected during routine diagnostics were used; however, informed consent was obtained from each volunteer who participated in the study. After examination of each volunteer by a dermatologist, demographic data, history and clinical signs/symptoms were recorded in a questionnaire. The skin was sterilized, and exudates were obtained from the suspected lesion border. For non-ulcerated lesions (nodules, papules or plaques) lesion scrapings were taken from the incision by a sterile scalpel.

In the clinical validation assay, a total of 49 patients were included in the study. Twenty-five lesion scraping/exudate samples were collected from CL suspected patients with different typical/atypical forms of the disease. Also, 24 fresh biopsies from skin lesions that mimic CL were taken.

Three reference strains of *Leishmania*, including *L. major* MRHO/IR/75/ER, *L. tropica* MHOM/SU/74/K27 and *L. infantum* MCAN/IR/96/LON49, were used as positive controls along with samples for PCR assay. Four reference strains of *L. major* including MRHO/IR/75/ER, MHOM/IL/81/Friedlin, MHOM/SU/73/5‐ASKH and MHOM/IR/2018/Q-MN, three reference strains of *L. tropica* including MHOM/IR/02/Mash10, MHOM/SU/74/K27 and MHOM/IR/2017/IPI, two reference strains of *L. infantum* including MCAN/IR/96/LON49 and MHOM/TN/80/IPT1 were used for LOD determination.

### Direct examination of stained smears

The lesion samples were smeared onto glass slides, air-dried, fixed with absolute methanol and stained with Giemsa. The slides were examined with a 40× and 100× immersion objectives under light microscope for the presence of amastigote forms at least 30 min before reporting the final result.

### Parasite culture

Part of the dermal exudate was inoculated under sterile conditions into the Novy-MacNeal-Nicolle (NNN) medium (Pasteur Institute, Tehran, Iran) overlaid with RPMI 1640 (Gibco Invitrogen, Carlsbad, CA, USA), incubated at 18–24 °C and the liquid phase was examined for parasite growth by light microscopy every other day for 6 weeks [25].

### DNA extraction

Additional lesion samples were taken and transferred to 2 ml vials containing sterile phosphate-buffered saline (PBS, pH 7.2) and used for DNA extraction. DNA extraction was carried out on each the lesion sample, culture isolates and *Leishmania* spp. reference strains using the QIAamp DNA Mini Kit (Qiagen, Hilden, Germany) which provides silica-membrane-based nucleic acid purification according to manufacturer’s instructions. DNA concentration and purity were estimated by measuring the absorbance at 260 nm and 280 nm using a NanoDrop one spectrophotometer (Thermo Fisher Scientific, Waltham, MA, USA). The DNA was kept at − 20 °C until further use.

### PCR assay set-up

Several primer sets were designed for the ITS region of the rRNA gene and those with acceptable physicochemical parameters were selected and aligned with the sequences from the databases using the nucleotide Basic Local Alignment Search Tool (BLASTn). BLASTn analysis enabled us to screen for possible non-specific interactions with other organisms or off-target amplification. BioEdit sequence alignment editor ver. 7.0.5.3 software (https://www.bioedit.com) and MEGA version 7.0.26 software [26] were used to align sequences of the ITS region from different strains of *Leishmania* to identify conserved and polymorphic regions for designing specific primers. The specific primer pairs which amplified the ITS2 region of different species of *Leishmania* with discriminating amplicon sizes, were selected.

PCR reactions were conducted in a total volume of 25 μl, containing 2× PCR buffer, containing Tris-HCl pH 8.5, 0.2% Tween 20, 3 mM MgCl_2_, 0.4 mM of each dNTP, 0.2 u/µl Ampliqon *Taq* DNA polymerase (Ampliqon, Odense, Denmark) and red dye for tracking. In addition, 0.2–0.5 μM of each specific primer pair UNIL-IR-P and UNIL-IR-M was used (Table 1). The DNA template concentrations were adjusted to a range of 0.01–100 pg/µl depending on the reaction. Annealing temperatures ranging between 54–62 °C were tested, and after several adjustments the temperature was optimized at 57.0 °C. Cycling conditions were as follows: initial denaturation 95 °C for 5 min; followed by 30 cycles of 95 °C for 30 s, 57  °C for 45 s, 72 °C for 45 s and a final extension step at 72 °C for 5 min. The amplified products were electrophoresed on 1.5% agarose gels in 1× Tris-acetate-EDTA (TAE) buffer. After performing electrophoresis, for DNA visualization, gels were stained with a 1:5000 dilution of the sensitive Eco-Stain Plus (Bio Basic Inc., Markham ON, Canada) in TAE buffer for 30 min. The results were visualized under UV light by using a Gel-Doc instrument (Vilber Lourmat, Collégien, France). The resolution of the images was improved for best quality results. The PCR conditions were optimized for each assay by using DNA from the three reference strains of *Leishmania* (*L. major* MRHO/IR/75/ER, *L. tropica* MHOM/SU/74/K27 and *L. infantum* MCAN/IR/96/LON49). The non-template negative controls (NTCs) were included for contamination control. Since the amplicons did not differ significantly in size, to confirm the results in uncertain cases a multiplex PCR assay was performed in the same tube using different primer pairs (Table 1) targeting kinetoplast DNA. Multiplex PCR conditions were established by using a mixture of DNA from all 3 *Leishmania* species. The 25 μl PCR reaction mixture consisted of Tris-HCl; pH 8.8, 0.01% Tween-20, 3 mM MgCl_2_, 0.5 mM each dNTPs, 3 U *Taq* DNA polymerase (Invitrogen, Carlsbad, CA, USA) and 0.5 μM of each of the three specific primer pairs. Cycling conditions were the same as above.

**Table 1:** Characteristics of PCR primer pairs

Strategy	Name	Species	Primer sequence (5′–3′)		GenBank ID	Nucleotide no.	Size (bp)
			Forward	Reverse			
Multiplex PCR	MULt-IR-P and MULt-IR-M	*L. tropica*	ACGCACCGCCTATACACAAA	ACTACTGCGTTCTTCACCGA	MH627386.1	138–292	155
	MULm-IR-P and MULm-IR-M	*L. major*	TCCGATGCTTACACCCCAAA	ATGCACGGGGATGACACAAT	KU680845.1	16–421	406
	MULi-IR-P and MULi-IR-M	*L. infantum*	ACATATACAACTCGGGGAGACC	AGGAAGCCAAGTCATCCATCG	KU975159.1	34–274	241
Universal PCR	UNIL-IR-P and UNIL-IR-M	*L. major*, *L. tropica*, *L. infantum*	CATGCCATATTCTCAGTGTCG	GGTCTGTAAACAAAGGTTGTCG	–	–	*L. m*: 740; *L. inf*: 690; *L. t*: 640

To avoid DNA contamination, different steps of the technical procedures were carried out in separate areas with dedicated consumables and decontamination. In lesion samples, the presence of possible PCR reaction inhibitors was ascertained by testing with specific primers for the *β*-actin gene. Some PCR products were subjected to sequencing for confirmation of correct amplification of the target.

### Optimization and limit of detection (LOD) determination

Following the development of the PCR protocols, to determine the LOD of the universal PCR for each species, DNA was extracted from promastigotes of 4 reference strains of *L. major*, 3 reference strains of *L. tropica* and 2 reference strains of *L. infantum.* To evaluate the possible interference of host DNAs, 30 ng of DNA purified from human skin cells were added to each dilution [27]. To determine LODs, 10-fold serially diluted concentrations including 100, 10, 1, 0.1, and 0.01 pg of parasite DNAs were spiked into the reactions then the PCR was performed with the same cycling program. The different possible combinations of the three species were also regarded and included in the PCR schedule. A total of 101 PCR reactions were performed along with NTCs.

### Validation with clinical specimens

#### Lesion scraping/exudate

Once the PCR conditions were optimized, the validity of identifying the correct species from clinical samples was assessed on skin lesions of CL patients caused by *L. major* or *L. tropica*. Lesion samples were subjected to universal PCR assay using *c.*10 ng of DNA per reaction. This amount of DNA was potentially a mixture of parasite and lesion cells naturally including the dermal/epidermal layer, infiltrated immune cells, bacterial flora and other local cells.

#### Fresh lesion biopsy

The validity of the universal PCR assay was tested with the biopsy samples obtained in sterile PBS from patients suffering from different cutaneous diseases which mimic CL, including fungal skin infections, lupus erythematosus, leprosy, skin neoplasm and tuberculosis.

### Performance of the test

For determining the performance of the universal PCR and to consider a case positive for CL, a combination of two molecular methods including a nested-PCR assay followed by RFLP using Rapid Digest *Mnl*I (Thermo Fisher Scientific, USA) was considered as reference (gold) standard [28] (see Additional file 1: Figure S1). Since this is a diagnostic test accuracy (DTA) study, a reference standard method was used to perfectly discriminate between participants with or without CL conditions and to provide unbiased estimates of the diagnostic accuracy measure of the index test (universal PCR). The authors were blinded of the result of testing on patients with skin lesions suggestive of CL. The gold standard method and universal PCR were made simultaneously on the same sample for each patient that was supposed to be included in the study; and the authors were not aware of the cases of CL while testing universal PCR.

In evaluation of universal PCR on lesion scraping/exudate samples, a positive result for one of conventional methods (smear or culture) was also considered. Sensitivity, specificity, positive predictive values (PPV), negative predictive values (NPV), and Cohen’s kappa measure of agreement (κ) were determined. The strength of agreement was defined as follows: poor (κ < 0.20); fair (κ of 0.21–0.40); moderate (κ of 0.41–0.60); good (κ of 0.61–0.80); and very good (κ of 0.81–1.00).

For the purpose of implementation in clinical settings of other laboratories, a few of either primer pairs alone or together with clinical specimens were submitted to three other centers of endemic area in Iran, where PCR mixes were produced locally and the tests were performed by different persons. The DNA was provided from either the same samples or was extracted from the samples of local CL patients referred for diagnostic purposes.

## Results

### Optimization and evaluation

**Fig. 1:**
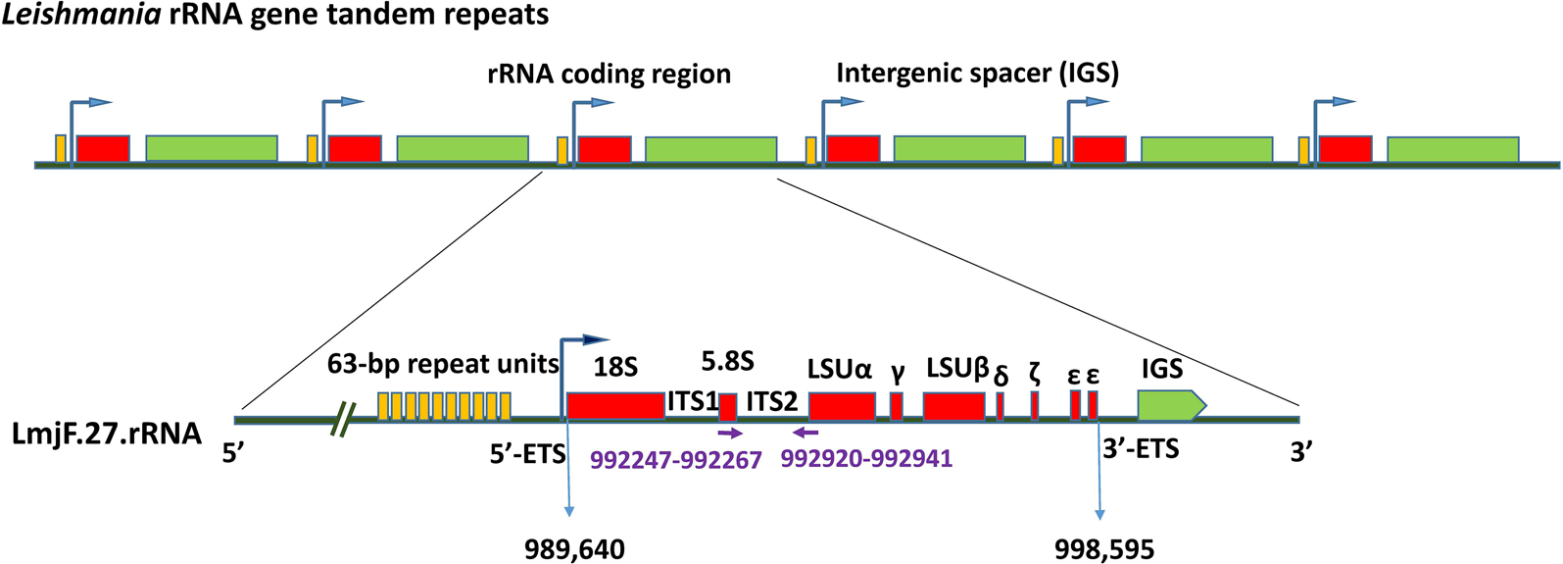
*Leishmania major* rRNA gene tandem repeats and primer positions. rRNA gene fragments and position of the universal primer pair in relation to the nucleotide sequences of chromosome 27 of *Leishmania major* strain Friedlin (TriTrypDB accession number: LmjF.27.rRNA), drawn to scale. The *18S*, ITS1, *5.8S*, ITS2 and *28S* units of rDNA are indicated. Small arrows show the forward and reverse primers. The start and end nucleotide of each primer are shown. Right angle arrows show transcription start sites. *Abbreviations:* LSU, large subunit; IGS, intergenic spacer; ETS, external transcribed spacer; ITS, internal transcribed spacer

The position of the universal primer pair related to the ITS2 region of the rRNA gene on chromosome 27 of *L. major* strain Friedlin is shown in Fig. 1. Also, sequence alignment of three CL causing species against Friedlin strain is included in Additional file 2: Alignment S1, showing the primer pair position and flanking sequences of ITS1, *5.8 S* and ITS2 rDNA fragments.

**Fig. 2:**
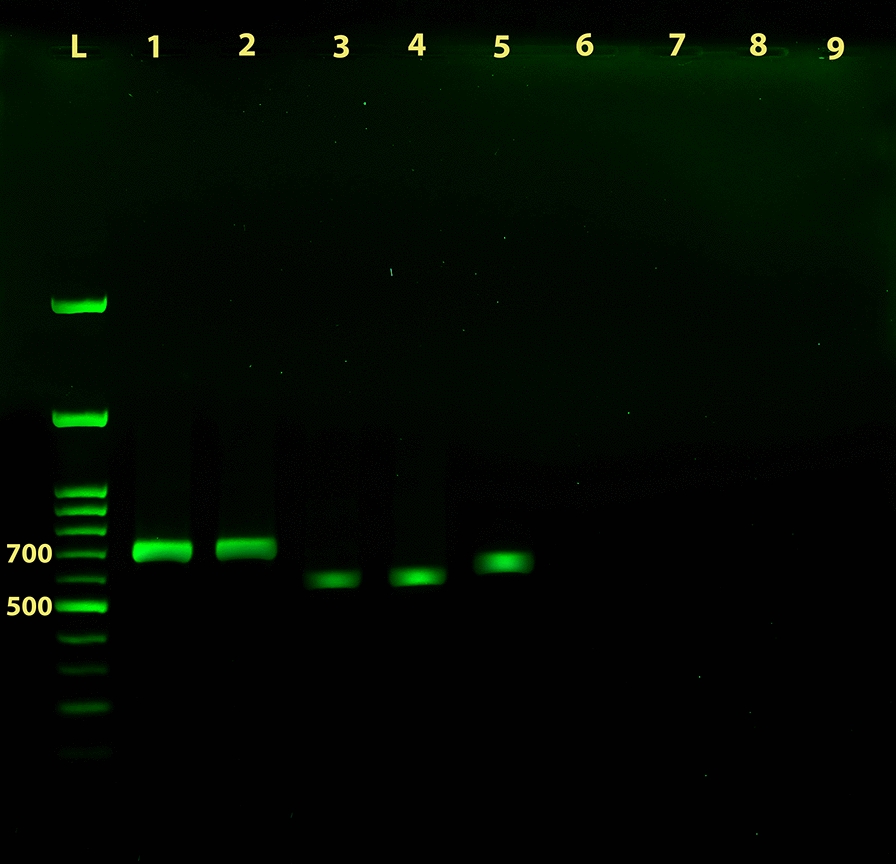
Identification of *Leishmania* species from biopsy samples. PCR amplification of *Leishmania* genomic DNA from fresh biopsy samples using the newly developed universal primers UNIL-IR-P and UNIL-IR-M subjected to electrophoresis on a 1.5% agarose gel. Lane 1: amplicon of *L. major* MRHO/IR/75/ER reference strain; Lane 2: amplicon of a positive *L. major* sample; Lane 3: amplicon of *L. tropica* MHOM/SU/74/K27 reference strain; Lane 4: amplicon of a positive *L. tropica* sample; Lane 5: amplicon of *L. infantum* MCAN/IR/96/LON49 reference strain; Lanes 6–8: negative biopsy samples that mimic CL, including fungal skin infection, tuberculosis and skin neoplasm, respectively. Lane 9: non-template negative control. Lane L: 100 bp DNA ladder

**Fig. 3:**
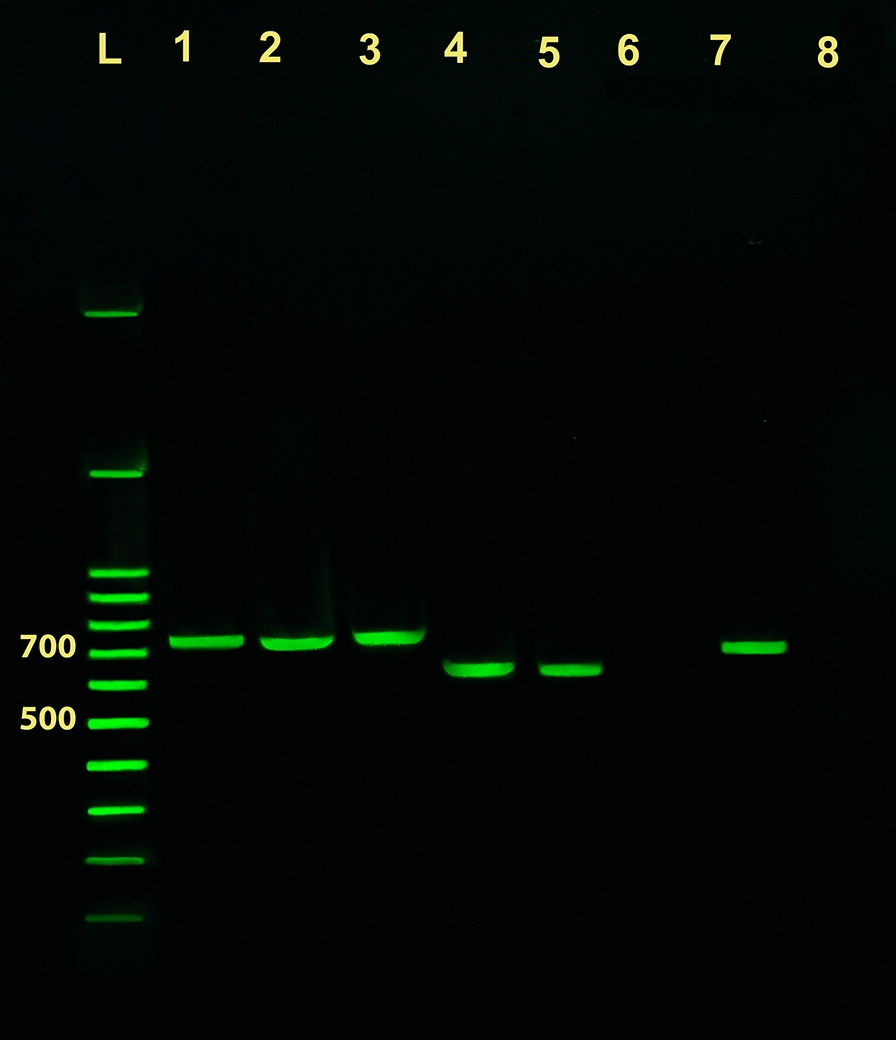
Identification of *Leishmania* species from scraping/exudate clinical samples. PCR amplification of *Leishmania* genomic DNA from lesion scraping/exudate using the newly developed universal primers UNIL-IR-P and UNIL-IR-M subjected to electrophoresis on a 1.5% agarose gel. Lane 1: amplicon of *L. major* MRHO/IR/75/ER reference strain; Lane 2: amplicon of *L. major* culture+/smear- samples; Lane 3: amplicon of *L. major* culture-/smear+ samples. Lane 4: amplicon of *L. tropica* MHOM/SU/74/K27 reference strain; Lane 5: amplicon of *L. tropica* culture+/smear+ samples; Lane 6: amplicon of culture-/smear-samples; Lane 7: amplicon of *L. infantum* MCAN/IR/96/LON49 reference strain; Lane 8: non-template negative control. Lane L: 100 bp DNA ladder

**Fig. 4:**
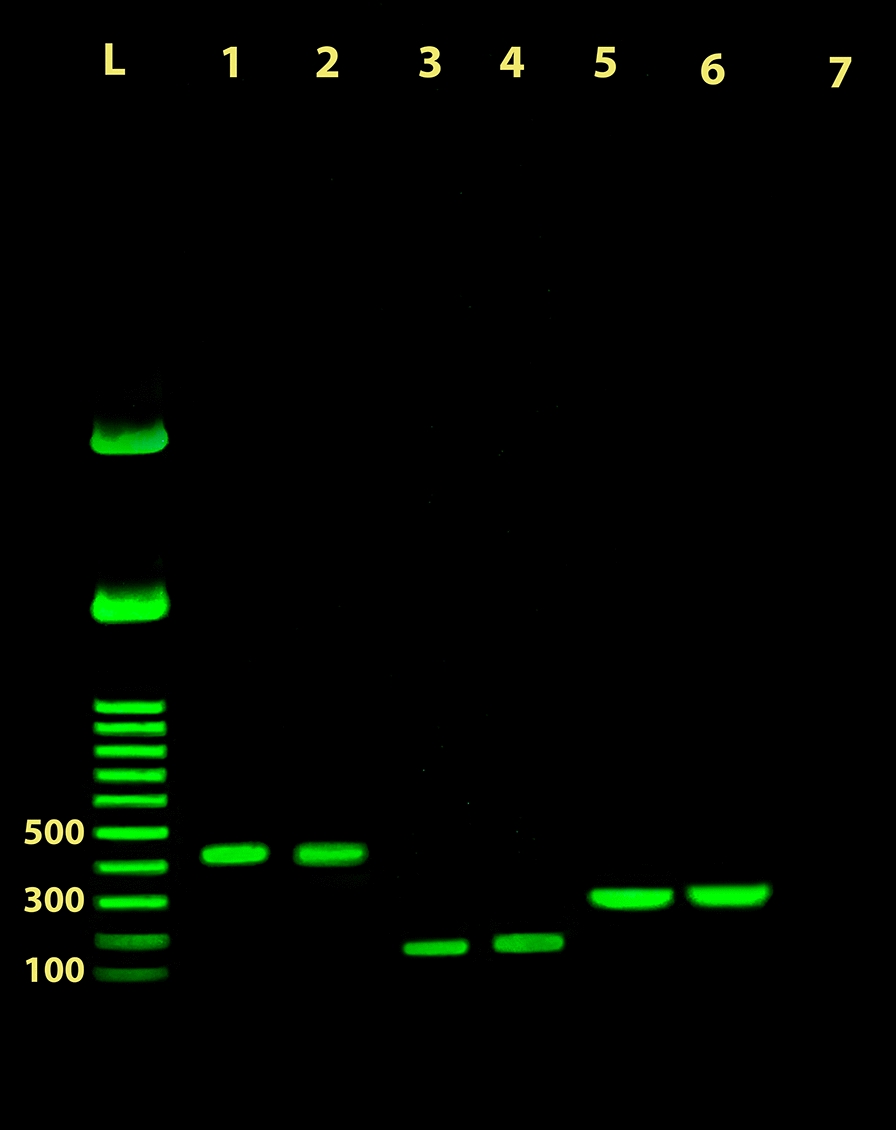
Multiplex PCR amplification of *Leishmania* genomic DNA from lesion samples using the newly developed primer pairs subjected to electrophoresis on a 1.0% agarose gel. Lane 1: amplicon of *L. major* MRHO/IR/75/ER reference strain; Lane 2: amplicon of a patient’s *L. major* isolate. Lane 3: amplicon of *L. tropica* MHOM/SU/74/K27 reference strain; Lane 4: amplicon of a patient’s *L. tropica* isolate; Lane 5: amplicon of *L. infantum* MCAN/IR/96/LON49 reference strain; Lane 6: amplicon of a patient’s *L. infantum* isolate. Lane 7: non-template negative control. Lane L: 100 bp DNA ladder

The amplicons were successfully obtained on scraping/exudate samples, fresh skin biopsy samples and promastigotes of reference strains as shown in Figs. 2, 3. During cycling set-up, in some instances weak non-specific products were also amplified at lower annealing temperatures which were then resolved at 57.0 °C. Figure 4 shows a representative result of multiplex PCR on samples.

Sequencing results showed that the primer sets and the PCR approach efficiently amplify the ITS2 locus of the rRNA gene of *Leishmania* species. The newly generated ITS2 sequences were submitted to the GenBank database under the accession numbers MN931857, MN931859 and MN969584.

This universal PCR was implemented successfully and validated for diagnosis of CL cases in three other laboratories of endemic area of Iran. No contamination of negative control was reported. In one CL-confirmed sample, the PCR tested negative but with increasing the amount of DNA the second PCR showed a positive result.

### LOD determination and applicability to culture promastigotes

**Fig. 5:**
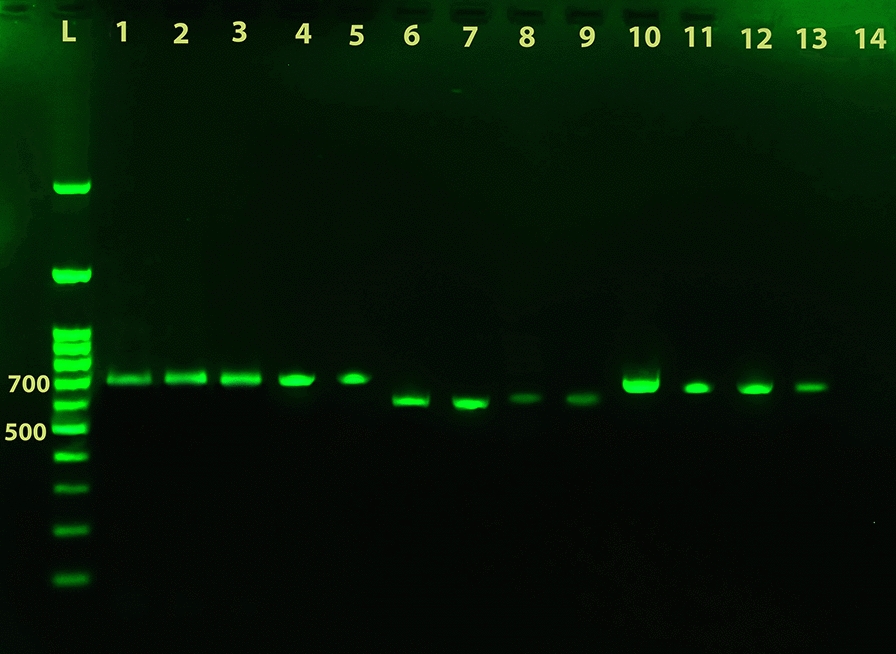
LOD determination based on PCR amplification of serially diluted DNA from culture promastigotes of *Leishmania* parasites. DNA was extracted from culture promastigotes of different reference strains of *L. major*, *L. tropica* and *L. infantum.* Serially 10-fold diluted amounts of 100, 10, 1, 0.1, and 0.01 pg of genomic DNAs were spiked into the reactions and the PCR amplification was performed as described with universal primers UNIL-IR-P and UNIL-IR-M. Human skin DNs were also added to each reaction for evaluation of possible interference. The different possible combinations of 3 species were also considered and included in the PCR schedule. PCR products were subjected to electrophoresis on a 1.5% agarose gel. Lanes 1–5: *L. major* DNA with 100 to 0.01 pg/µl concentrations, respectively; Lanes 6–9: *L. tropica* DNA with 100 to 0.1 pg/µl concentrations, respectively; Lanes 10–13 *L. infantum* DNA with 100 to 0.1 pg/µl concentrations, respectively; Lane 14: non-template negative control. Lane L: 100 bp DNA ladder

The universal PCR showed the potential to amplify as little as 0.01 pg of DNA from promastigotes of *L. major* and 0.1 pg of DNA from promastigotes of *L. tropica* and *L. infantum*, 0.1 pg is about half a parasite’s genome (Table 2). This detection limit was unaffected by the presence of DNA from other *Leishmania* species in a mixed reaction or by the presence of host DNAs (Fig. 5). For some DNA samples, the detection limit was higher, up to 1 pg; variations were probably caused by DNA degradation.

**Table 2:** Result of universal PCR assay on serially diluted amount of DNA from promastigotes of *Leishmania* reference strains

Species	Frequency of positive tests, *n* (%)					
	100 pg/µl	10 pg/µl	1 pg/µl	0.1 pg/µl	0.01 pg/µl	NTC
*L. major* (*n* = 4)	4 (100)	45 (100)	4 (100)	4 (100)	4 (100)	0 (0)
*L. tropica* (*n* = 3)	3 (100)	3 (100)	3 (100)	3 (100)	3 (100)	0 (0)
*L. infantum* (*n* = 2)	2 (100)	2 (100)	2 (100)	2 (100)	0 (0)	0 (0)
*L. major* *+* *L. tropica* (*n* = 4)^a^	4 (100)	4 (100)	4 (100)	4 (100)	4 (100)	0 (0)
*L. major* *+* *L. infantum* (*n* = 3)^a^	3 (100)	3 (100)	3 (100)	3 (100)	0 (0)	0 (0)
*L. tropica* *+* *L. infantum* (*n* = 3)^a^	3 (100)	3 (100)	3 (100)	3 (100)	0 (0)	0 (0)

^a^Presence of both bands of two species has been regarded as positive

*Abbreviation*: NTC: non-template control

### Validation on clinical samples

Tables 3 and 4 list the demographic and clinical information of the patient volunteers included in the study for blinded validation of the PCR on their lesion samples. In total, 25 scraping/exudate samples and 24 skin tissue biopsy samples were included in the validation assay, both using *c.*10 ng of DNA.

**Table 3:** Clinical information of CL suspected patients and the result of laboratory tests on lesion scraping/exudate specimens

No.	Code	Sex	Age	Country	Travel to endemic area	*Leishmania* spp.	Type of CL	Treatment history	Lesion location	No. of lesions	Size of lesions (mm)^a^	Laboratory tests results		
												Smear microscopy	NNN culture	Universal PCR
1	HOMAGH	M	34	Iran	No	*L. major*	Multiple lesion	Topical ketoconazole	Left leg	3	15 × 15	Neg	Pos	Pos
2	ZAPAAL	F	4	Iran	No	*L. major*	Typical local	No	Left leg	1	5 × 5	Neg	Pos	Pos
3	MODAHA	M	18	Iran	No	*L. major*	Typical local	No	Left hand	1	30 × 25	Neg	Pos	Pos
4	MAGOAL	F	31	Iran	No	*L. major*	Typical local	No	Left leg	1	22 × 20	Neg	Pos	Pos
5	EZTEGH	M	62	Iran	No	*L. major*	Typical local	IL Glucantime	Leg	1	60 × 45	Neg	Neg	Pos
6	ZASAMO	F	52	Iran	No	*L. major*	Sporotrichoid	IL Glucantime	Right leg	1	60 × 45	Pos	Neg	Pos
7	MOSEHA	M	3	Iran	No	*L. major*	Typical local	No	Right ear	1	11 × 5	Pos	Neg	Pos
8	MOALJA	M	8	Iran	No	*L. major*	Multiple lesion	Topical gentamycin	Left and right leg	3	12 × 12	Pos	Neg	Pos
9	ALYAPA	M	50	Iran	No	*L. major*	Multiple lesion	No	Left leg	3	5 × 5	Pos	Neg	Pos
10	ABLOKH	M	38	Iran	No	*L. major*	Multiple lesion	No	Left and right leg	2	25 × 25	Pos	Neg	Pos
11	ZOKHZA	F	35	Iran	No	*L. major*	Multiple lesion	No	Left leg	2	30 × 35	Pos	Pos	Pos
12	EBKAAZ	M	21	Afghanistan	Yes- Iran	na	Multiple lesion	IM Glucantime	Neck and left hand	4	50 × 15	Neg	Neg	Neg
13	MOLOAH	M	23	Iran	No	na	Typical local	No	Left hand	1	20 × 12	Neg	Neg	Neg
14	JAKAHA	M	55	Iran	No	na	Typical local	No	Left hand	1	50 × 55	Neg	Neg	Neg
15	ALSEEB	M	28	Iran	No	*L. major*	Disseminated	No	Trunk, hand	11	15 × 15	Pos	Pos	Pos
16	ZAKEFA	F	62	Iran	No	*L. major*	Multiple lesion	Cryotherapy	Left hand	3	15 × 20	Pos	Pos	Pos
17	ZIMAUN	F	44	Iran	Yes-Iraq	*L. major*	Typical local	No	Hand	1	30 × 35	Pos	Pos	Pos
18	NAJAUN	M	40	Iran	No	*L. tropica*	Typical local	No	Head and face	1	25 × 25	Pos	Pos	Pos
19	SOGHAM	F	35	Iran	NO	*L. major*	Typical local	No	Right hand	1	17 × 5	Pos	Pos	Pos
20	BANAKH	M	18	Afghanistan	Yes- Iran	*L. tropica*	Typical local	No	Neck	1	50 × 15	Pos	Pos	Pos
21	MOPOAH	M	57	Iran	No	*L. major*	Multiple lesion	No	Left and right hand	5	2 × 15	Pos	Pos	Pos
22	MATEMO	M	22	Iran	Yes- Iran	*L. major*	Typical local	No	Right hand	1	18 × 10	Pos	Pos	Pos
23	ABPASA	M	55	Iran	No	*L. major*	Typical local	No	Right hand	1	30 × 18	Pos	Pos	Pos
24	KORAFA	F	57	Iran	No	*L. major*	Multiple lesion	No	Right hand–Left leg	2	8 × 8	Pos	Pos	Pos
25	FAKOHA	F	15	Iran	No	*L. tropica*	Typical local	No	Head and face	1	17 × 5	Pos	Pos	Pos

^a^If multiple lesions were present all lesions were measured and a mean size was calculated and presented

*Abbreviations*: IL, intralesional injection; IM, intra muscular injection; Pos, positive; Neg, negative; na, not applicable

**Table 4:** The result of universal PCR assay on biopsy samples collected from patients with skin diseases mimic CL

No.	Code	Age	Sex	Geographical region	Travel to endemic area	Location	Type	Gold standard result	Universal PCR
1	KHHA	65	F	Iran (Kordestan)	No	Right calf	na	Neg	Neg
2	GOBA	92	M	Iran (Rey)	No	Foot toes	na	Neg	Neg
3	BAMO	48	F	Iran (Rey)	Yes (Iran: Kermanshah)	Lower abdomen	na	Neg	Neg
4	ZADO	58	F	Iran (Karaj)	No	Arm	na	Neg	Neg
5	HOAJ	49	M	Iran (Karaj)	No	Forearm	na	Neg	Neg
6	FANO	23	F	Iran (Ghods)	Yes (Iran: Mashhad)	Left foot	na	Neg	Neg
7	MAHA	39	M	Iran (Tehran)	No	Cheek	na	Neg	Neg
8	ALHA	69	M	Iran (Tehran)	No	Forearm	na	Neg	Neg
9	RAES	39	M	Iran (Eslamshahr)	No	Ear	na	Neg	Neg
10	SOEB	59	M	Iran (Ghods)	No	Right and left hand	na	Neg	Neg
11	MOHO	38	M	Iran (Tehran)	No	Right hand	na	Neg	Neg
12	RAAH	16	M	Iran (Tehran)	No	Right hand	*L. major*	Pos	Pos
13	ATSH	6	F	Iran (Tehran)	Yes (Iran: Aghaliabbas)	Left hand	*L. major*	Pos	Pos
14	ABGH	27	M	Iran (Tehran)	No	Head and face	*L. tropica*	Pos	Pos
15	FAMO	20	F	Iran (Tehran)	No	Right leg		Pos	Pos
16	EBES	42	M	Iran (Tehran)	No	Left hand	*L. tropica*	Pos	Pos
17	BIDE	69	M	Iran (Eslamshahr)	Yes (Iran: Azerbaijan)	Leg	*L. major*	Pos	Pos
18	DAAH	48	M	Iran (Mazandaran)	Yes (Iran: Isfahan, Yazd, Kerman)	Arm	*L. major*	Pos	Pos
19	FAPI	3	M	Iran (Ardabil)	Yes (Iraq: Karbala)	Head and face	*L. major*	Pos	Pos
20	YAMO	42	M	Iran (Tehran)	Yes (Iran: Bandarabbas)	Trunk	*L. major*	Pos	Pos
21	MOFE	26	M	Iran (Karaj)	Yes (Iraq: Karbala)	Hand	*L. major*	Pos	Pos
22	BEMO	65	M		No	Head and hand	*L. tropica*	Pos	Pos
23	MAMA	40	F	Iran (Tehran)	Yes (Iran: Ardabil)	Right hand	*L. major*	Pos	Pos
24	ASMA	12	F	Iran (Tehran)	Yes (Iran: Isfahan)	Face, hand, leg	*L. major*	Pos	Pos

*Abbreviation*: na, not applicable; Pos, positive; Neg, negative

As shown in Table 3, patients had different clinical forms of CL, such as sporotrichoid, disseminated and multiple lesions. Nearly 40.0% of the cases developed more than one lesion, with the hand as the most common site of lesion onset (52.0%). The mean size (± standard deviation, SD) of CL lesions was 22.6 ± 14.18 mm. Some patients received topical or systemic treatment which might influenced the test results. The mean age (± SD) of the patients was 34.68 ± 18.39 years. Some patients had history of travel to endemic areas in Iran, Afghanistan or Iraq during the last 6 months before lesion onset.

Table 4 shows the main information of the CL suspected patients enrolled for diagnosis by fresh biopsy sampling. The mean age (± SD) of the patients was 41.46 ± 22.29 years. Hand was the most common site of lesion onset (37.5%).

Figure 2 depicts one representative result of PCR assays on skin tissue biopsy samples and Fig. 3 depicts one representative result on scraping/exudate clinical samples. In samples containing *L. major*, occasionally, a weak PCR product was amplified in the assay, but this product showed a faint and smaller than 500 bp amplicon which was clearly below the actual product size of *L. major* samples. No products were amplified using samples obtained from patients suffering from other skin diseases that mimic CL.

### Performance of the test

The performance of universal PCR on scraping/exudate and skin biopsy samples are abbreviated in Table 5. *Leishmania* spp. were successfully identified in the various CL skin samples, as 22 of 22 scraping/exudate samples and 13 of 13 biopsy samples showed a positive *Leishmania* product of the correct size with this PCR assay. No false negative PCR reaction occurred and the sensitivity of the PCR on scraping/exudate specimens was 100% (95% confidence interval, CI: 84.56–100%), the same as that of the biopsy samples (95% CI: 75.29–100%). There was no false positive result and the specificity of PCR on scraping/exudate (95% CI: 71.51–100%) and biopsy (95% CI: 29.24–100%) samples was 100%.

**Table 5:** Performance of conventional parasitology tests and universal PCR in the diagnosis of CL cases

Method	Sensitivity (%)^a^	95% CI	Specificity (%)^b^	95% CI	PPV (%)^c^	NPV (%)^d^
Conventional parasitology						
Stained smear	77.27 (17/22)	54.63–92.18	100 (3/3)	29.24–100	100 (17/17)	37.50 (3/8)
NNN culture	72.73 (16/22)	49.78–89.27	100 (3/3)	29.24–100	100 (16/16)	33.33 (3/9)
Universal PCR						
On lesion scraping/exudate	100 (22/22)	84.56–100	100 (3/3)	29.24–100	100 (22/22)	100 (3/3)
On lesion biopsy	100 (13/13)	75.29–100	100 (11/11)	71.51–100	100 (13/13)	100 (11/11)

^a^No. of true positive/no. of infected persons

^b^No. of true negative/no. of non-infected persons

^c^No. of true positive/no. of positive results

^d^No. of true negative/no. of negative results

*Abbreviations*: CI, confidence intervals; PPV, positive predictive value; NPV, negative predictive value

Smear microscopy and parasite culture alone detected 77.27% (95% CI: 54.63–92.18%) and 72.73% (95% CI: 49.78–89.27%) of the positive CL specimens, respectively, while culture and microscopy together improved the overall sensitivity to more than 90% (21/22). The specificity of both conventional methods was 100% (95% CI: 29.24–100%).

The agreement between PCR and smear microscopy was moderate (*κ *= 0.45; *P *= 0.007) and between PCR and culture was fair (*κ *= 0.39; *P *= 0.014) and the overall agreement between the PCR and parasitological approaches was very good (*κ *= 0.83; *P* < 0.0001), when the results of the parasitology tests were determined by considering both smear and culture results.

## Discussion

Among different genetic markers used for *Leishmania* identification, kinetoplast DNA (kDNA) and the ITS region have been vastly used to detect the parasite in different biological samples [18, 20, 29–31]. While highly sensitive approaches for identifying particular *Leishmania* species have been described, if multiple *Leishmania* spp. need to be differentiated in a diagnostic laboratory, molecular approaches that require different PCR primers for each species have the potential for carryover contamination. Although strategies to minimize this potential risk have been developed, contamination has been observed even when strict protocols were followed [29]. Moreover, using multiple primers is not cost-effective, needs more materials for PCR reactions, and more time to set-up the procedure. Our goal was to develop a molecular approach for species level discrimination that requires only one pair of PCR primers. We focused on the ITS region: the rRNA internal spacers are subject to less evolutionary pressure and show more sequence divergence than the coding regions and have been proposed as targets for molecular typing. We designed species-specific primers flanking the ITS2 region adequate for our diagnostic goal to differentially identify *Leishmania* spp. causing CL. The substantial differences in the ITS2 region spanned by these primers are due to InDel and polymorphic loci of repeated motifs including microsatellite markers, typically with one to six non-coding nucleotides per repeated unit [8, 20]. However, we did not access to samples positive for *L. aethiopica* to compare the sensitivity of the primer pairs.

The classical methods have limitations, especially with regard to sensitivity. In this study the sensitivity of either microscopy or parasite culture alone was less than 78%, while culture and microscopy together improved the overall sensitivity to more than 90% (21/22 positive samples). In other studies, the sensitivity of conventional parasitological methods in the diagnosis of CL ranged from ~30% to ~85% for either smear or culture and were always lower than molecular methods based on PCR [32, 33].

In this study, PCR of the ITS2 target enabled identification of 100% of the CL patients by analysis of DNA from positive samples including scraping/exudate (25/25) and biopsy samples (24/24), which included strains of *L. major* and *L. tropica*. Since neither conventional parasitology nor PCR showed false positives, all the assays were 100% specific. In support of our approach in selection of the ITS region as an amplification target, previous studies have reported high sensitivity of the ITS2-PCR in diagnosis of CL and visceral leishmaniasis (VL) cases, as compared to microscopic examination [18]. Also, the real-time PCR method for the ITS2 region in *Leishmania* has been suggested as one of the most sensitive diagnostic tests for identifying parasite load [34, 35]. In another report, the kDNA PCR showed the highest sensitivity (98.7%) compared to any other assay, followed by the rRNA ITS PCR (91.0% sensitivity) [36].

In our study, there were ten discordant results among 25 scraping/exudate samples including four samples with positive culture which were negative by smear examination, and five samples which were positive by microscopy but negative in culture growth. One sample of a CL patient that was negative in both culture and examination by light microscopy, was confirmed positive by the universal PCR. This patient received intralesional Glucantime and the negative result following conventional parasitological approaches might be attributed to disrupted amastigote remnants in lesion specimens. All ten samples which were negative in one or both of parasitological approaches were identified as *L. major* by the universal PCR approach. We were blind of the result of “gold standard” testing on skin lesions suggestive of CL, so the authors were not aware of the cases of CL while testing the universal PCR on samples. False negative results highlight the underestimation of identification of CL cases which needs to be regarded in clinical management of the disease.

One advantage of PCR-based molecular approaches in diagnosis of infections is that they do not need a viable organism for detection. In some instances, negative culture results might be attributable to fastidious or non-viable parasites in the specimens collected for culture or to contamination of the NNN media. On the other hand, the lack of a positive smear for some of the culture/PCR positive samples might be due to unequal distributions of parasites in the lesion, such that the parasite number in the portion collected for smear preparation was not enough for detection by microscopy. Nevertheless, proper detection of amastigotes requires an experienced microscopist.

With 100% PPV and NPV by PCR, a positive result from either lesion scraping/exudate or biopsy sample should always be considered a true positive, and a negative test result should always be considered a true negative.

Molecular diagnosis provides improvements in clinical diagnosis of leishmaniasis; however, the selection of the most suitable PCR technique is not always easy. We have validated our PCR on both lesion scraping/exudate and skin biopsy samples. Some developed PCRs did not show enough sensitivity in clinical settings and were recommended only for identification of *Leishmania* on culture isolates [37]. CL lesions due to *L. infantum* contain a lower parasitic load than those due to *L. tropica* or *L. major* and is reportedly more difficult to isolate. Therefore, dermal aspirates of the lesion might not be sufficient for microscopic detection of *L. infantum* infection but it should be suitable for PCR diagnosis [38].

We used culture promastigotes of reference strains to determine LOD. As shown here, the universal PCR could detect as little as 0.1–0.01 pg of *Leishmania* DNA and shows a higher sensitivity than certain other species- and genus-specific PCRs developed to detect *Leishmania* species causing CL [29].

Simultaneous co-infection with two species of *Leishmania* in clinical cases of leishmaniasis is not implausible [39], therefore the possibility of mixed infection of different strains should be regarded in diagnostics approaches. We have examined the possible interference of the presence of DNA from other *Leishmania* species in the PCR reaction and the efficiency of the universal PCR was not affected by co-infections.

Diagnostic tools are necessary to detect and identify *Leishmania* species both in endemic areas where different species coexist and in non‐endemic areas where imported CL cases occur due to travel and population migration [40]. In this study, CL cases due to *L. major* or *L. tropica* were also detected among immigrants of Afghanistan and Iraq, two main immigrant populations who have a long history of living in Iran. Afghanistan is one of the major foci of CL in the world and the capital Kabol is the endemic area with highest incidence rate of ACL in the world, with an estimated annual incidence of 67,500 cases. We used the ITS region of the rRNA gene of *Leishmania* as a target to develop an affordable, specific, and easy-to-use PCR test to discriminate *L. infantum*, *L. tropica* and *L. major*. This universal PCR assay enables identification the infecting *Leishmania* species in both lesion exudate and biopsy samples, without any cross-reaction with other skin diseases compatible with CL. It has also the capability to be used as a tool to characterize *Leishmania* species on culture promastigotes or directly on cryopreserved samples.

## Conclusions

Molecular techniques have significantly improved the diagnosis of CL-suspected patients in endemic areas of leishmaniasis. The presented universal PCR assay can be used routinely in diagnosis of suspected CL cases, since the performance of this rDNA-ITS2 PCR to reliably identify *L. major*, *L. tropica* and *L. infantum* is validated on clinical samples. However, additional studies with more clinical samples from areas of CL endemicity in the Old World are needed to evaluate the feasibility of using this universal PCR in clinical settings.

## Supplementary information

**Additional file 1: Figure S1.** The results of electrophoresis of the products of the nested PCR-RFLP based amplification of DNA extracted from *Leishmania* reference strains before and after enzymatic digestion with *Mnl*I. Digestion was performed by adding 5 U of *Mnl*I restriction enzyme to a 13 μl aliquot of the nested PCR product for 3 h at 37 °C and the products were visualized on 2.5% agarose gel electrophoresis. Lanes 1–3: *L. major* (245 bp), *L. tropica* (99 bp) and *L. infantum* (200 bp) before digestion, respectively. Lane 4: products of *L. major* (106, 73 and 44-bp fragments); Lane 5: products of *L. tropica* (75, 67 and 19-bp fragments); Lane 6: products of *L. infantum* (127, 33 and 30-bp fragments) after enzymatic digestion. Lane 7: negative control. Lane L: 100 bp ladder.

**Additional file 2: Alignment S1.** Nucleotide sequence alignment of the rDNA-ITS region of CL-causing species of *Leishmania*. *Leishmania* sequences generated by ITS2-PCR using universal primers UNIL-IR-P and UNIL-IR-M. Sequences from three different strains of CL-causing *Leishmania* spp. are aligned against *L. major* (Friedlin strain). The primer pair position and flanking sequences are shown. The yellow highlighted positions indicate the start and end of *5.8S* and green highlighted position indicates initiation of LSUα of *28S* fragments. The primer binding regions are shown in rectangles.

## Data Availability

Data supporting the conclusions of this article are included within the article and its additional files. The datasets used and/or analysed during the present study are available from the corresponding author on reasonable request.

## Abbreviations

CL: cutaneous leishmaniasis
ACL: anthroponotic CL
ZCL: zoonotic CL
PCR: polymerase chain reaction
ITS: internal transcribed spacer
ETS: external transcribed spacer
NNN: Novy-MacNeal-Nicolle
NTC: non-template negative control
LOD: limit of detection
kDNA: kinetoplast DNA
VL: visceral leishmaniasis
