# Supplementary material for: Performance of a universal PCR assay to identify different Leishmania species causative of Old World cutaneous leishmaniasis
Source: Parasit Vectors. 2020 Aug 27;13:431. doi: 10.1186/s13071-020-04261-5 (PMC7450935; doi:10.1186/s13071-020-04261-5)
Supplement: Supplementary file 1 — Additional file 1: Figure S1. The results of electrophoresis of the products of the nested PCR-RFLP based amplification of DNA extracted from Leishmania reference strains before and after enzymatic digestion with MnlI. Digestion was performed by adding 5 U of MnlI restriction enzyme to a 13 μl aliquot of the nested PCR product for 3 h at 37 °C and the products were visualized on 2.5% agarose gel electrophoresis. Lanes 1–3: L. major (245 bp), L. tropica (99 bp) and L. infantum (200 bp) before digestion, respectively. Lane 4: products of L. major (106, 73 and 44-bp fragments); Lane 5: products of L. tropica (75, 67 and 19-bp fragments); Lane 6: products of L. infantum (127, 33 and 30-bp fragments) after enzymatic digestion. Lane 7: negative control. Lane L: 100 bp ladder. [file 13071_2020_4261_MOESM1_ESM.docx]

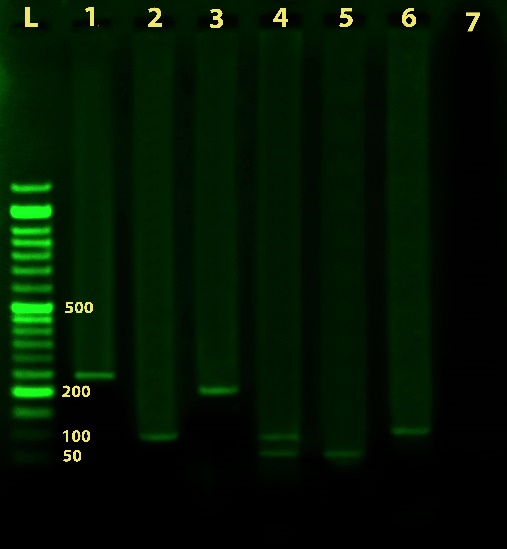


**Additional file 1: Figure S1- The results of the electrophoresis of the products of the nested PCR-RFLP based amplification of DNA extracted from *Leishmania* reference strains before and after enzymatic digestion by *MnlI*.**
