# Supplementary material for: Performance of a universal PCR assay to identify different Leishmania species causative of Old World cutaneous leishmaniasis
Source: Parasit Vectors. 2020 Aug 27;13:431. doi: 10.1186/s13071-020-04261-5 (PMC7450935; doi:10.1186/s13071-020-04261-5)
Supplement: Supplementary file 2 — Additional file 2: Alignment S1. Nucleotide sequence alignment of the rDNA-ITS region of CL-causing species of Leishmania. Leishmania sequences generated by ITS2-PCR using universal primers UNIL-IR-P and UNIL-IR-M. Sequences from three different strains of CL-causing Leishmania spp. are aligned against L. major (Friedlin strain). The primer pair position and flanking sequences are shown. The yellow highlighted positions indicate the start and end of 5.8S and green highlighted position indicates initiation of LSUα of 28S fragments. The primer binding regions are shown in rectangles. [file 13071_2020_4261_MOESM2_ESM.docx]

**
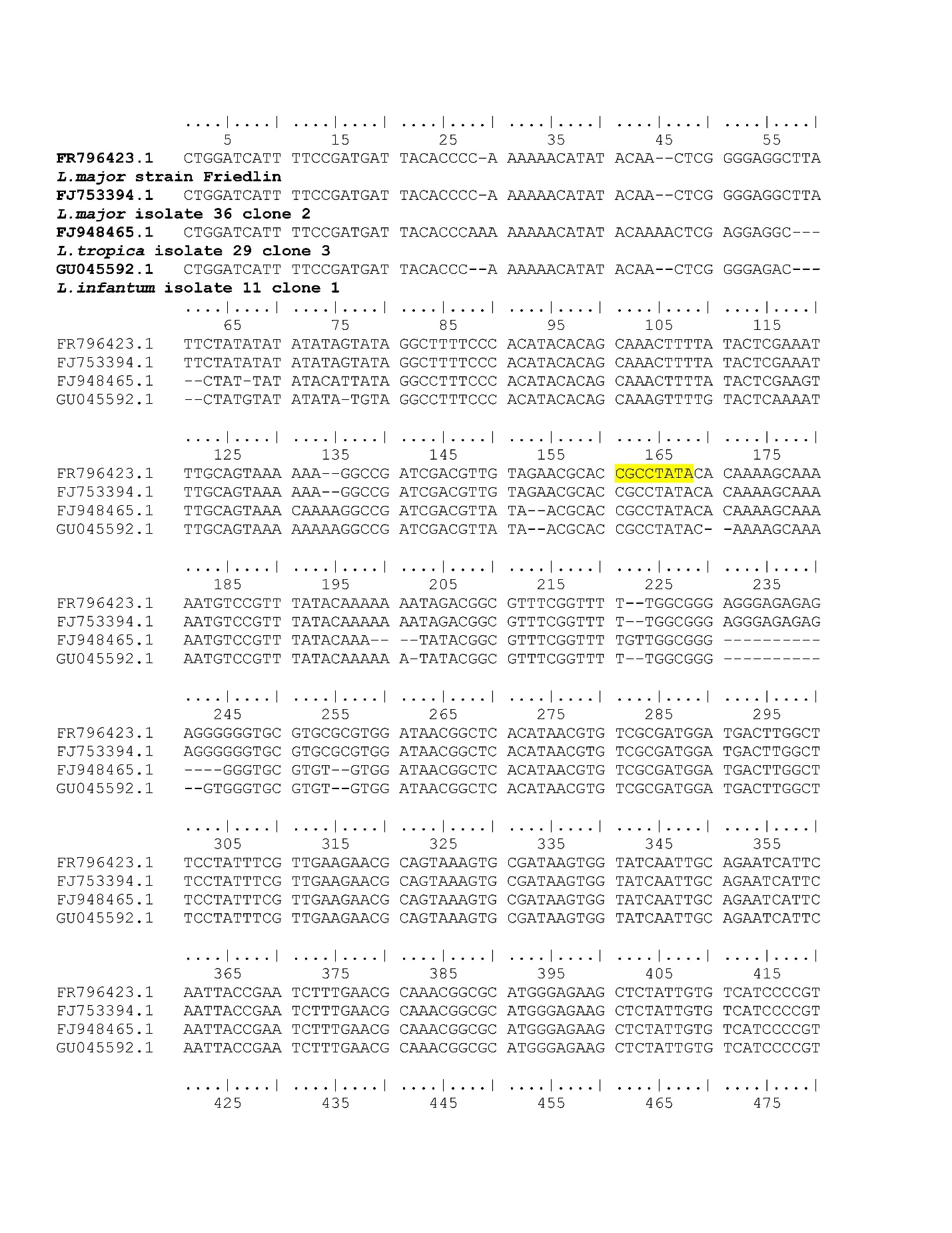
** **
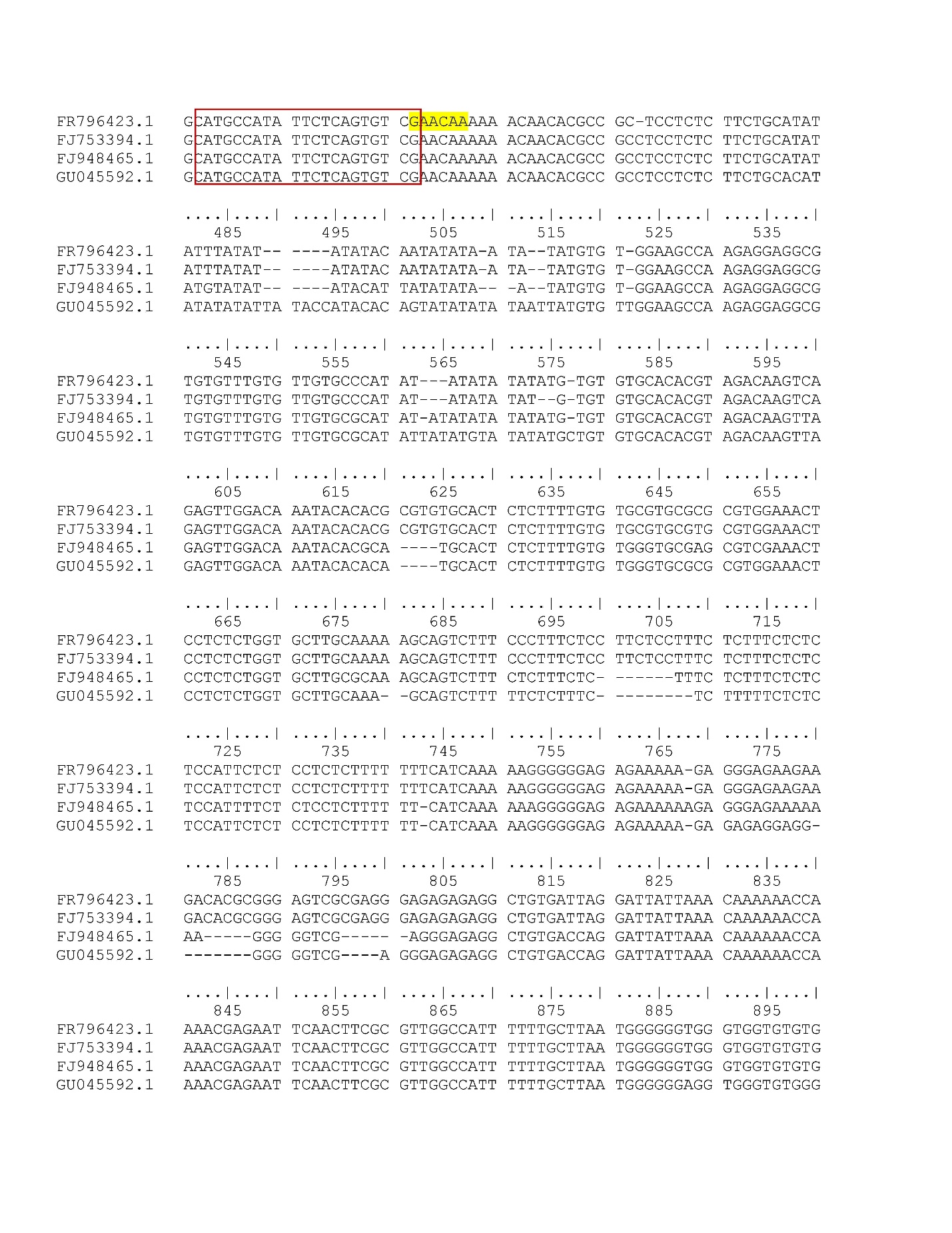
**
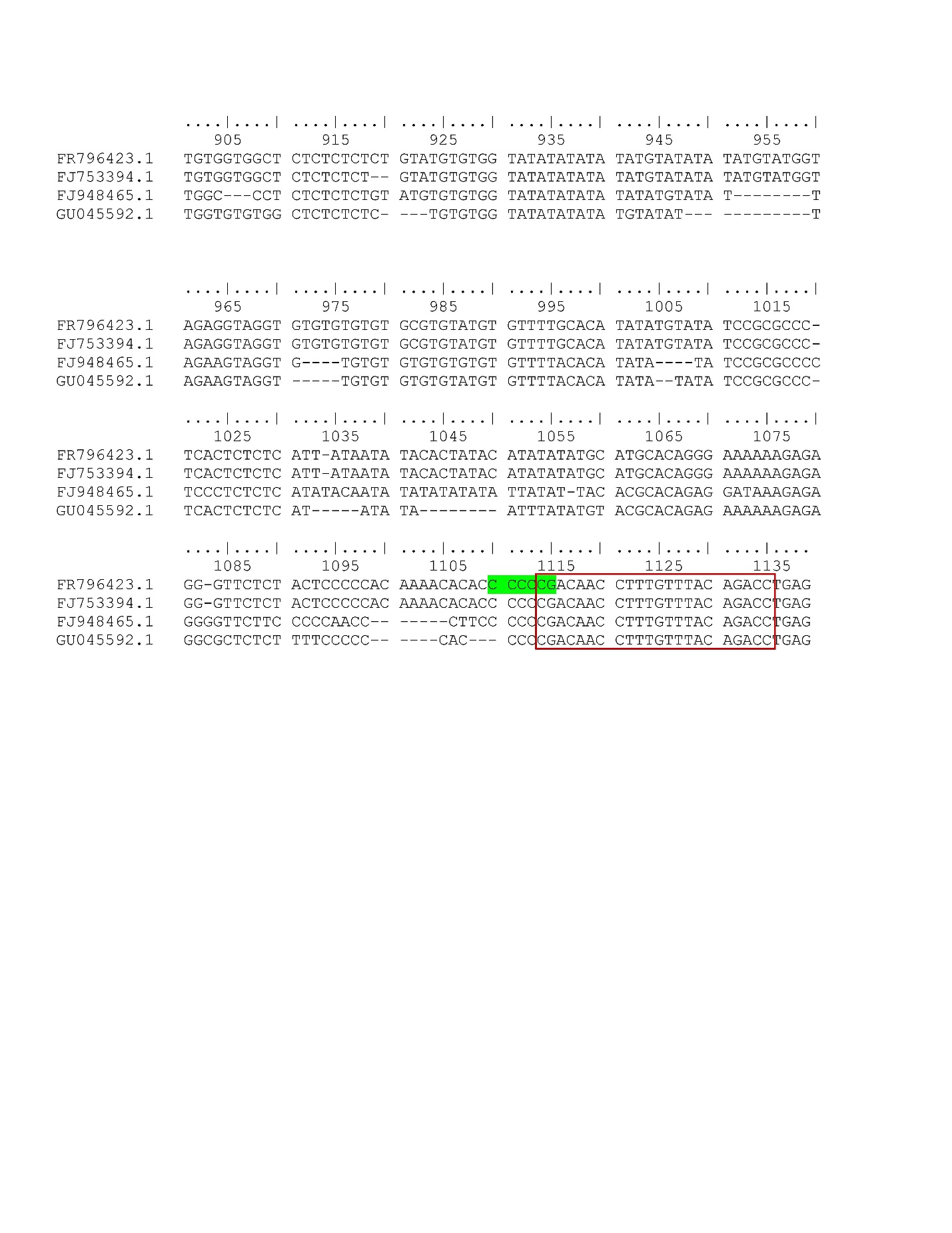
 **Additional file 2: Alignment S1- Nucleotide sequence alignment of rDNA-ITS region of CL causing species of *Leishmania* parasites**
